# The effect of proprioceptive vestibular rehabilitation on sensory-motor symptoms and quality of life

**DOI:** 10.1055/s-0045-1806832

**Published:** 2026-01-28

**Authors:** Partiksha, Sakshi Sadhu

**Affiliations:** 1 Lovely Professional University, Lovely School of Allied Medical Sciences, Department of Physiotherapy, Phagwara PB, India.

Dear Editor,

We read with interest a recently-published article titled “The Effect of Proprioceptive Vestibular Rehabilitation on Sensory-Motor Symptoms and Quality of Life” 1 in the journal *Arq. Neuro-Psiquiatr.* 2024;82(11):s00441790568 that has piqued our interest. Here are important methodological and interpretive issues that need to be addressed even if the study offers insightful information on the advantages of proprioceptive vestibular rehabilitation (PVR) for peripheral vestibular hypofunction (PVH).

First, despite being characterized as randomized and single-blind, the study's design raises concerns about possible bias. Only thirty people were split up into three groups, which seems like a small sample size for interpreting findings. Although the authors admit that attrition occurs for several reasons, they neglected to conduct an intention-to-treat analysis, which could have a substantial impact on the results.

Second, the authors use statistical significance ( *p* < 0.05) to support their claim that PVR is superior. 2 The lack of impact estimates for group comparisons, however, prohibits readers from determining the data's clinical significance. For example, although group 1 demonstrated notable gains in functional mobility and balance, it is yet unknown if these variations are important in everyday situations.

Furthermore, it is admirable but understudied to include qualitative outcomes like sensory processing patterns. Although they do not go into great detail about the processes behind this effect, the authors imply that proprioceptive stimulation enhances sensory integration. The theoretical underpinnings of their results and the findings' practical relevance are weakened by this omission.

The study's scope is further constrained by the lack of long-term follow-up. For clinicians creating intervention protocols, vestibular rehabilitation's sustainability is crucial, particularly when dealing with a chronic illness like PVH. Potential regression or long-term improvement after the intervention is not taken into consideration by the eight-week length.

Finally, even though the discussion section makes an effort to tie the findings to earlier research, several findings are not well supported. In vestibular rehabilitation, for instance, the claim that proprioceptive stimulation “replaces the missing vestibular input” oversimplifies the intricate neuroplasticity at action.
